# Rare presentation of BRASH syndrome with hypoglycemia and altered mental status

**DOI:** 10.1186/s12245-023-00517-w

**Published:** 2023-07-03

**Authors:** Marián Sedlák, Kamila Brúsiková, Vladimíra Sobolová, Michal Králik

**Affiliations:** 1 Emergency Medicine Department, Faculty of Medicine and Louis Pasteur University Hospital, Pavol Jozef Safarik University in Kosice, Kosice, Slovakia; 2 Internal Medicine Department, Faculty of Medicine and Louis Pasteur University Hospital, Pavol Jozef Safarik University in Kosice, Kosice, Slovakia; 3 Urology Department, Faculty of Medicine and Louis Pasteur University Hospital, Pavol Jozef Safarik University in Kosice, Kosice, Slovakia; 4 Internal Department, AGEL Hospital Kosice-Saca, Kosice, Slovakia

**Keywords:** BRASH, Hypoglycemia, Bradycardia, Hyperkalemia

## Abstract

**Background:**

BRASH syndrome (bradycardia, renal failure, atrioventricular nodal blockade, shock, and hyperkalemia) is a rare clinical condition with potentially severe outcomes. Patients with BRASH syndrome can present with diverse signs and symptoms and are usually in critical condition, but if recognized early, the syndrome is treatable and may have a favorable prognosis.

**Case presentation:**

This case study presents a 74-year-old patient with a history of multiple chronic conditions who was brought to the emergency department with a suspected cerebrovascular accident, altered mental status, and bradycardia. A head computed tomography scan was unremarkable but laboratory results showed hyperkalemia, acidosis, and renal failure with concomitant progressive hypoglycemia. The patient was diagnosed with a BRASH syndrome characterized by a vicious cycle of atrioventricular nodal blockade induced by the potentiated effect of beta-blockers or calcium channel blockers, in combination with progressive hypoglycemia due to the suspected accumulation of anti-diabetic medications, which influenced the presentation and initial triage in the emergency department. She was admitted to the intensive care unit for further management, where she continued to improve and was ultimately discharged in a relatively stable condition.

**Conclusion:**

This case study highlights the importance of considering rare and atypical presentations of medical conditions, particularly in elderly patients who may have multiple comorbidities. Early recognition and prompt management of such cases are crucial for improving patient outcomes.

## Background

BRASH syndrome (bradycardia, renal failure, atrioventricular (AV) nodal blockade, shock, and hyperkalemia) is an uncommon and relatively new clinical entity. The condition can be summarized as a vicious cycle of AV nodal blockade, usually induced by beta-blockers or calcium channel blockers, which subsequently leads to bradycardia, decreased tissue perfusion, shock, renal failure, worsening of cardiac output, hyperkalemia, and acidosis, which further worsen and fuel the abovementioned pathophysiological processes. Although patients with BRASH syndrome are usually in critical condition and may suffer serious consequences including death, if recognized early, the condition is treatable and may have a favorable prognosis [1, 2].

## Case presentation

In this case study, we present a 74-old female patient, who was brought to the emergency department (ED) by emergency medical services (EMS) with a suspected stroke. She complained of sudden generalized weakness, her speech deteriorated, and her ability to move was severely limited. The patient was reported to have had a last known normal of 3.5 h before the admission. In addition to a history of a cerebrovascular accident (CVA) 2 years ago (with residual minor global aphasia), her chronic conditions included metabolic syndrome, arterial hypertension, obesity (BMI 50,78), insulin-dependent type 2 diabetes mellitus (DM) with chronic microvascular organ complications, chronic venous insufficiency, chronic heart failure (NYHA 4), chronic kidney disease (G1A2 KDIGO), and permanent atrial fibrillation with left anterior fascicular block. Her medications included rivaroxaban, carvedilol, perindopril, urapidil, atorvastatin, amlodipine, eplerenone, furosemide, empagliflozin, insulin, potassium tablets, and vitamins.

**Fig. 1:**
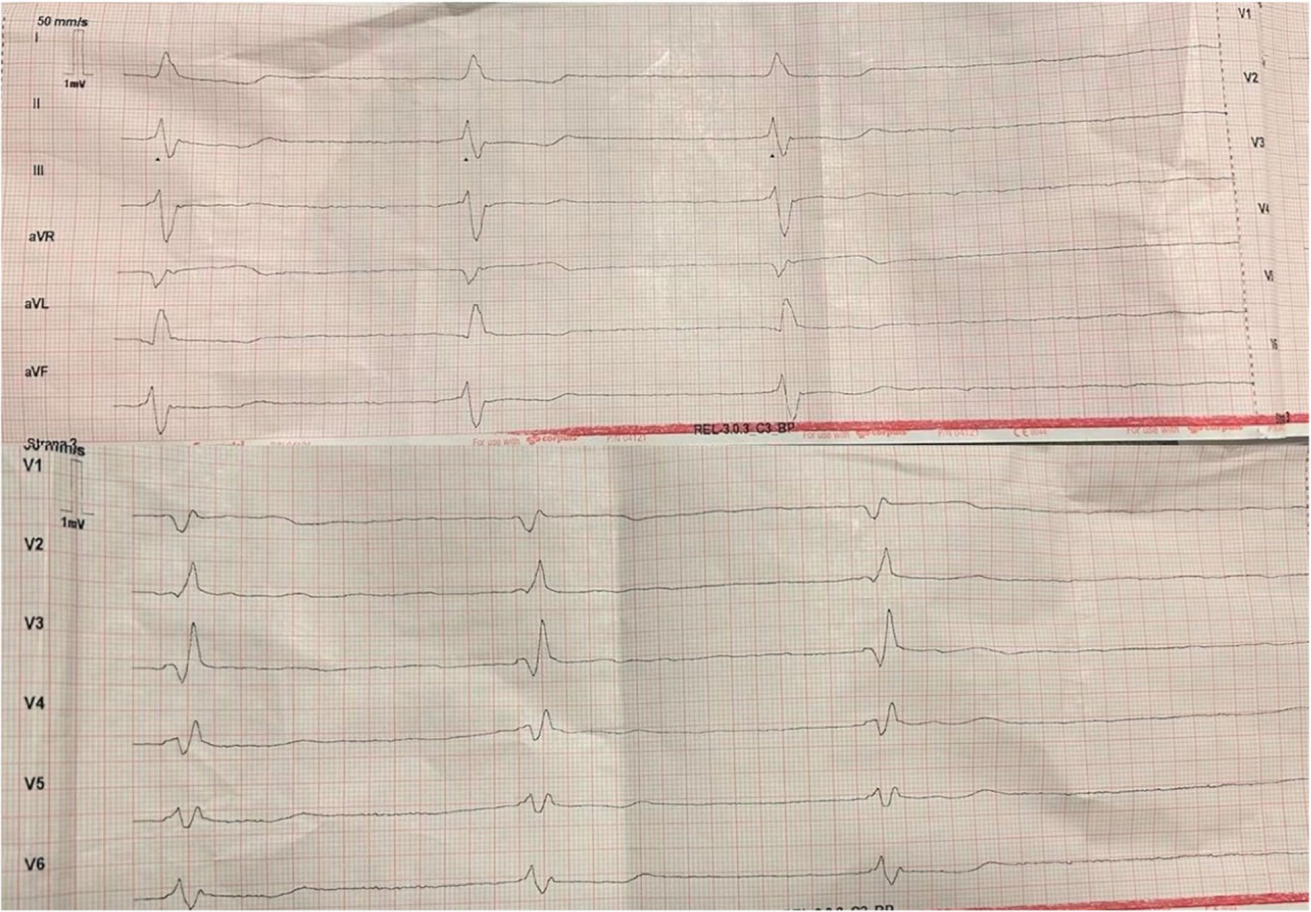
ECG obtained by emergency medical services showing atrial fibrillation with a right bundle branch block and slow ventricular response, heart rate 30 beats/min

On clinical examination by the EMS crew, she presented with altered mental status with a Glasgow Coma Scale (GCS) of 14 (4–4-6) with significant dysarthria, patent airways, blood pressure (BP) of 170/100 mmHg, respiratory rate of 20 breaths/min, SpO2 95% on room air, glycemia 91.8 mg/dL, and heart rate of 31 beats/min (bpm) (Fig. 1). EMS administered 1 mg of Atropine with a heart rate increase to 45 bpm. She was transferred to the ED with a suspected CVA and symptomatic bradycardia.

**Fig. 2:**
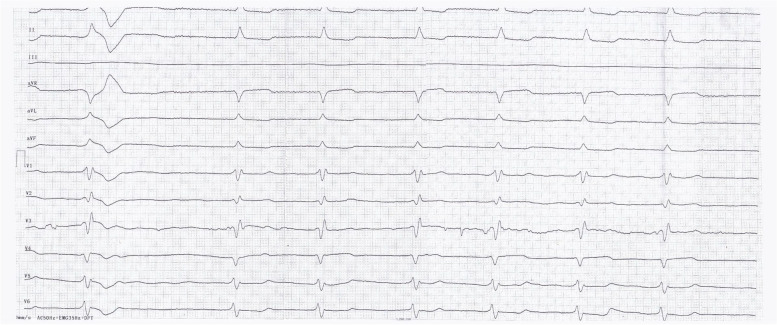
ECG obtained at the emergency department showing atrial fibrillation with a right bundle branch block and slow ventricular response, heart rate 45 beats/min after administration of 1 mg of Atropine

On arrival at the ED, her clinical examination was as follows: her airway was patent, SpO2 was 93%, repeated blood pressure measurements on both hands read 70/30 mmHg, and heart rate of 40–45 bpm with atrial fibrillation with a slow ventricular response (Fig. 2). Her neurological exam confirmed altered mental status with a Glasgow Coma Scale of 14 (4–4–6) with significant dysarthria and bilateral pupil miosis with poor photoreaction but no other focal neurological deficit (NIHSS 10 points). She denied acute pain, dyspnea, and possible intoxication or misuse of medications, but she admitted a possibility of recent overuse of NSAIDs due to chronic pain in the past few weeks.

She was placed on a cardiac monitor and her labs were drawn. She was immediately started on a dopamine drip at 10 mcg/kg/min with a heart rate response of 50–55 bpm but with only a slight increase in blood pressure. She received fluids and her dopamine drip was titrated to 15 mcg/kg/min. Due to a suspected CVA, she underwent an ED neurological examination, and subsequently, she was referred to computed tomography (CT) imaging, which did not confirm any intracranial pathology. Laboratory findings included potassium 5.8 mEq/L, blood urea nitrogen (BUN) 104.17 mg/dL, creatinine 5.58 mg/dL, serum albumin 2.98 g/dL, pH 7.136, and serum bicarbonate 11.3 mEq/L. Her repeated glycemia was 43.2 mg/dL. Other laboratory findings were unremarkable.

Intravenous glucose was administered to correct hypoglycemia. Calcium gluconate and furosemide were administered. Due to concomitant hypoglycemia, insulin was deferred. The patient was admitted to the intensive care unit (ICU) for further management in relatively stable condition with GCS 15, BP 108/61 mmHg, and a heart rate of 70 bpm (on continuous dopamine infusion). In the ICU, she was placed on a norepinephrine drip in addition to dopamine due to persistent hypotension. Rehydration and complex supportive therapy were initiated and optimal diuresis was achieved by furosemide. Vasopressors were stopped the next day, and she was eventually downgraded to the ward while she continued to improve clinically. Her potassium, glucose, and renal function values improved to her baseline levels while on intravenous fluids with diuresis supported by furosemide. She was ultimately discharged after 12 days in the hospital with outpatient follow-up recommendations. Her insulin treatment was adjusted and slightly decreased, her beta-blocker treatment was discontinued, and she remained on a low dose of calcium channel blockers in addition to the rest of her chronic medications.

## Discussion and conclusions

BRASH syndrome is a clinical entity with a convoluted pathophysiology and clinical picture. Although the pathophysiology underlying BRASH syndrome has been established since the 1990s, its recognition as a specific entity is very recent and little is known regarding its true epidemiology [2]. The initiation of the vicious cycle of BRASH syndrome (bradycardia, renal failure, atrioventricular nodal blockade, shock, and hyperkalemia) follows several pathways. Identified triggers include mostly hypovolemia and dehydration, acute kidney injury, the potentiated effect of medications, or generally any event promoting hyperkalemia or renal failure [1–3]. Older patients with multiple comorbidities, mainly cardiac and renal diseases, may be at higher risk of developing BRASH, especially if their medications include multiple different AV-nodal blocking medications [2]. The symptomatology of BRASH is rather complicated. Patients may present with a variety of signs and symptoms including symptomatic bradycardia, syncope, generalized weakness, altered mental status, dyspnea or dizziness/lightheadedness, and others, which makes differential diagnosis of a patient presenting with any BRASH syndrome signs and symptoms very challenging [1]. However, early recognition and diagnosis are critical to avoid multiorgan failure and adverse outcomes [2]. To date, there were several case studies of BRASH syndrome reported [1]. Only a few complex articles and reviews broadly analyzed the condition, its epidemiology, and treatment. Such a complex presentation of BRASH syndrome complicated by altered mental status, suspected stroke, and hypoglycemia (due to accumulation and potentiation of antidiabetic medications) has yet to be reported.

Our patient was transferred to the ED by EMS with a complex of symptoms, which were evaluated by EMS and neurologist as a possible stroke event, given the patient’s complaints, clinical presentation, and personal history. Bradycardia was initially identified and the treatment by atropine was attempted; however, it was initially assigned to a potential complex of stroke symptoms (suspected Cushing reflex). The combination of a non-focal neurological exam, head CT scan negative for intracranial hemorrhage, and angiography negative for large vessel occlusion prompted the team to look into differential diagnoses to explain her complex symptomatology. Laboratory findings uncovered mild hyperkalemia, elevated BUN, creatinine, and serum bicarbonate with concomitant hypoglycemia.

We can speculate that neurologic symptomatology and altered mental status were present due to the low-flow state causing brain hypoperfusion and the manifestation of the brain’s “locus minoris resistentiae” in combination with acute renal failure and subsequent uremia. Hypoglycemia and decreased brain perfusion may very well imitate stroke symptoms too, but her glycemia was repeatedly measured by the EMS crew on the scene and she was not severely hypoglycemic at that time. Patients with advanced chronic kidney disease and insulin use have a higher risk for serious hypoglycemic events [4]. Metabolism and the effect of insulins and other medications used to treat diabetes mellitus during BRASH cascade are not well understood. Although the first measurement on the scene could not be classified as hypoglycemia, the repeated measurement confirmed very low values, which can cause neurological impairment. As a severe diabetes patient, she was on numerous hypoglycemic medications. The patient denied overuse of medications or improper dosing protocols. We can speculate if acute renal failure in combination with bradycardia, hypoperfusion, and accumulation of medications and their metabolites can cause synergic effects and subsequently reinforce hypoglycemic effects of antidiabetic medications even without overdose or incorrect dosing, as seen in this particular case.

Hyperkalemia may precipitate cardiac rhythm changes including bradycardia or even heart block, asystole, ventricular tachycardia, and ventricular fibrillation. However, these changes usually occur with more severe hyperkalemia ((K +) > 7.0 mmol/L); however, the exact level of hyperkalemia which can cause these changes varies considerably [5]. Severe hyperkalemia often has additional ECG findings, such as QRS widening and typical changes to the morphology of the P and T-waves, which are often absent in BRASH syndrome [2]. ECG and rhythm abnormalities that are disproportionate to the degree of hyperkalemia should suggest other possible factors that may be influencing and accentuating the effects of mildly elevated potassium levels [6].

The treatment of BRASH syndrome is primarily focused on resuscitation and stabilization of vital functions, mostly with vasopressors and fluids, with careful treatment of other conditions as well. One of the common errors in managing BRASH syndrome is focusing only on a single component of the syndrome, rather than approaching it from its complex perspective [2, 7]. This may happen when BRASH is not recognized (e.g., we are treating the patient only within the silo of one of its specific symptoms) or one particular symptom seems to be more dangerous than another, which is often ignored in the emergency care phase (e.g., profound bradycardia). An important part of care is adjustments to chronic medications upon discharge to decrease the risk of future recurrence.

Ultimately, BRASH syndrome remains an under-recognized clinical diagnosis [1, 6]. Improving understanding of diverse presenting signs and symptoms and the influence of other acute and chronic conditions can help healthcare workers in faster recognition and better management of this syndrome, ultimately improving survival and patient outcomes. Further research is needed to create and establish effective triaging tools, consistent diagnostic criteria, and therapeutic guidelines to reduce the time until appropriate treatment, unnecessary interventions, and complications related to its complicated pathophysiology and presentation.

## Data Availability

All data generated or analyzed during this study are included in this published article.

## Abbreviations

BMI: Body mass index
BP: Blood pressure
BPM: Beats per minute
BRASH: Bradycardia, renal failure, atrioventricular nodal blockade, shock, and hyperkalemia
BUN: Blood urea nitrogen
CT: Computed tomography
DM: Diabetes mellitus
ED: Emergency department
EMS: Emergency medical services
GCS: Glasgow Coma Scale
ICU: Intensive care unit
KDIGO: Kidney Disease Improving Global Outcomes
NIHSS: National Institutes of Health Stroke Scale
NSAID: Non-steroidal anti-inflammatory drugs
NYHA: New York Heart Association Functional Classification
